# Mechanism of Rifampicin Inactivation in *Nocardia farcinica*

**DOI:** 10.1371/journal.pone.0162578

**Published:** 2016-10-05

**Authors:** Heba Abdelwahab, Julia S. Martin Del Campo, Yumin Dai, Camelia Adly, Sohby El-Sohaimy, Pablo Sobrado

**Affiliations:** 1 Department of Biochemistry, Virginia Tech, Blacksburg, VA, 24061, United States of America; 2 Department of Chemistry, Faculty of Science, Damietta University, Damietta, 34517, Egypt; 3 Fralin Life Science Institute, Virginia Tech, Blacksburg, VA, 24061, United States of America; 4 Department of Food Technology, Arid Lands Cultivation Research Institute, City of Scientific Research and Technological Applications, Alexandria, 21934, Egypt; 5 Virginia Tech Center for Drug Discovery, Virginia Tech, Blacksburg, VA, 24061, United States of America; National Institutes of Health, UNITED STATES

## Abstract

A novel mechanism of rifampicin (Rif) resistance has recently been reported in *Nocardia farcinica*. This new mechanism involves the activity of rifampicin monooxygenase (RifMO), a flavin-dependent monooxygenase that catalyzes the hydroxylation of Rif, which is the first step in the degradation pathway. Recombinant RifMO was overexpressed and purified for biochemical analysis. Kinetic characterization revealed that Rif binding is necessary for effective FAD reduction. RifMO exhibits only a 3-fold coenzyme preference for NADPH over NADH. RifMO catalyzes the incorporation of a single oxygen atom forming an unstable intermediate that eventually is converted to 2′-N-hydroxy-4-oxo-Rif. Stable C4a-hydroperoxyflavin was not detected by rapid kinetics methods, which is consistent with only 30% of the activated oxygen leading to product formation. These findings represent the first reported detailed biochemical characterization of a flavin-monooxygenase involved in antibiotic resistance.

## Introduction

*Nocardia spp*. are Gram-positive bacteria that cause a lung infection, known as pulmonary nocordiosis, in immuno-compromised and immuno-competent individuals [1]. Rifampicin (Rif) is a member of the rifamycin family of antibiotics used in combinational therapy for the treatment of nocardiosis and numerous mycobacterial infections, including tuberculosis and leprosy [1, 2]. Rif inhibits protein biosynthesis by binding to the β-subunit of the DNA-dependent RNA polymerase (RNAP), blocking RNA transcription in bacteria [3]. However, the emergence of Rif resistance has limited its prolonged use in the treatment for nocardiosis [4]. For this reason, different approaches have been considered, such as semi-synthetic Rif modifications, which leads to decreased toxicity and shortened treatment duration [5]. In addition, investigation of the Rif resistance mechanism(s) has been elucidated and this knowledge may help diminish the rate of antibiotic resistance [5–9]. In *Nocardia farcinica* IFM 10152, one of the mechanisms of Rif resistance has been shown to originate via the emergence of mutations in the *rpoB2* gene, a duplicated mutant copy of the *rpoB* gene, which encodes for the β-subunit of RNAP [10, 11]. These mutations occur through substitutions at positions that influence Rif binding, which attenuates its antibiotic activity. In addition, enzyme-catalyzed Rif modifications that lead to resistance have been reported in *Nocardia sp*. and closely related bacteria. For example, enzymatic modifications through phosphorylation [12], glycosylation [13], and ribosylation [14, 15], which target the polyketide backbone in Rif have been reported. These modifications may lead to a decrease in the binding affinity to the β-subunit of RNAP [11, 16]. In *N*. *farcinica*, Rif degradation has also been reported [11]. Rif degradation was later shown to be due to the presence of the *rox* gene, which encodes for rifampicin monooxygenase (RifMO) [11, 17].

**Fig 1:**
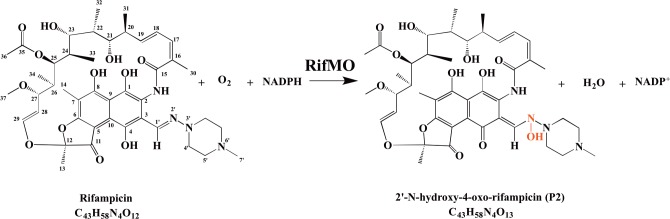
Reaction catalyzed by RifMO.

RifMO, a flavin-dependent enzyme, was proposed to initiate Rif decomposition by catalyzing the conversion of Rif to 2′-N-hydroxy-4-oxo-Rif (Rif-OH) in the presence of NADPH and oxygen (Fig 1). The product was found to exhibit ~ 100-fold lower antibiotic activity against *Kocuria rhizophila*, *Staphylococcus aureus*, and *Bacillus subtilis* [11]. The final inactivation product of this pathway has never been characterized; it was only referred to as a decolorized product [8, 9, 11]. Previous studies in *Rhodococcus erythropolis* and *Mycobacterium smegmatis* revealed changes in Rif’s visible region absorbance spectrum that were accompanied by its color change and a decline in its bacteriostatic activity [8, 9, 11]. Although, these studies have suggested that Rif decomposition occurs through modification by RifMO [8, 9, 11], no kinetic or structural characterization of RifMO enzymes is currently available. Here, we present the heterologous expression, purification, and detailed biochemical characterization of RifMO.

## Results

### Protein expression and purification

**Fig 2:**
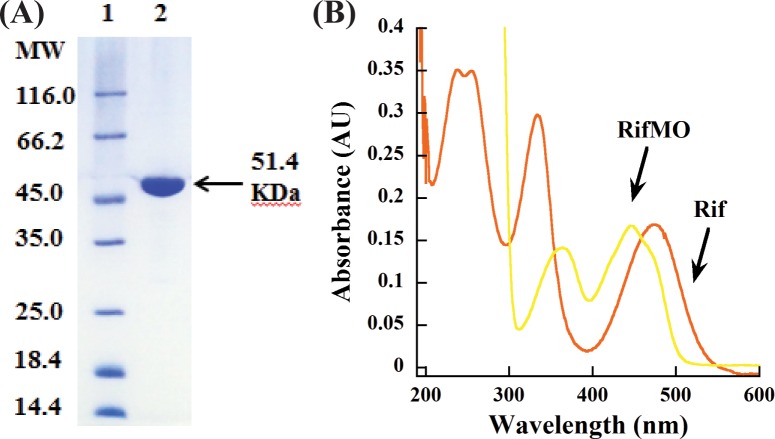
(A) SDS-PAGE of purified RifMO. Lane 1, Molecular weight markers; lane 2, final RifMO sample. (B) UV-visible spectrum of 15 μM Rif and RifMO. The flavin spectrum shows absorbance maxima at 366 nm and 449 nm.

RifMO was expressed as an *N*-terminus-6xHis-fusion protein in pET15b. The 6xHis-tag was cleaved with thrombin and the final yield was ~85 mg of purified RifMO per six liters of growth media (Fig 2A). The purified RifMO contained ~ 95% flavin incorporation. Spectra characteristic to flavin-containing enzymes were observed with absorbance maxima at 366 and 450 nm (Fig 2B). An extinction coefficient of 10,990 M^-1^cm^-1^ at 450 nm was calculated based on comparison with liberated free FAD.

### Rif binding to oxidized RifMO and spectral changes during catalysis

**Fig 3:**
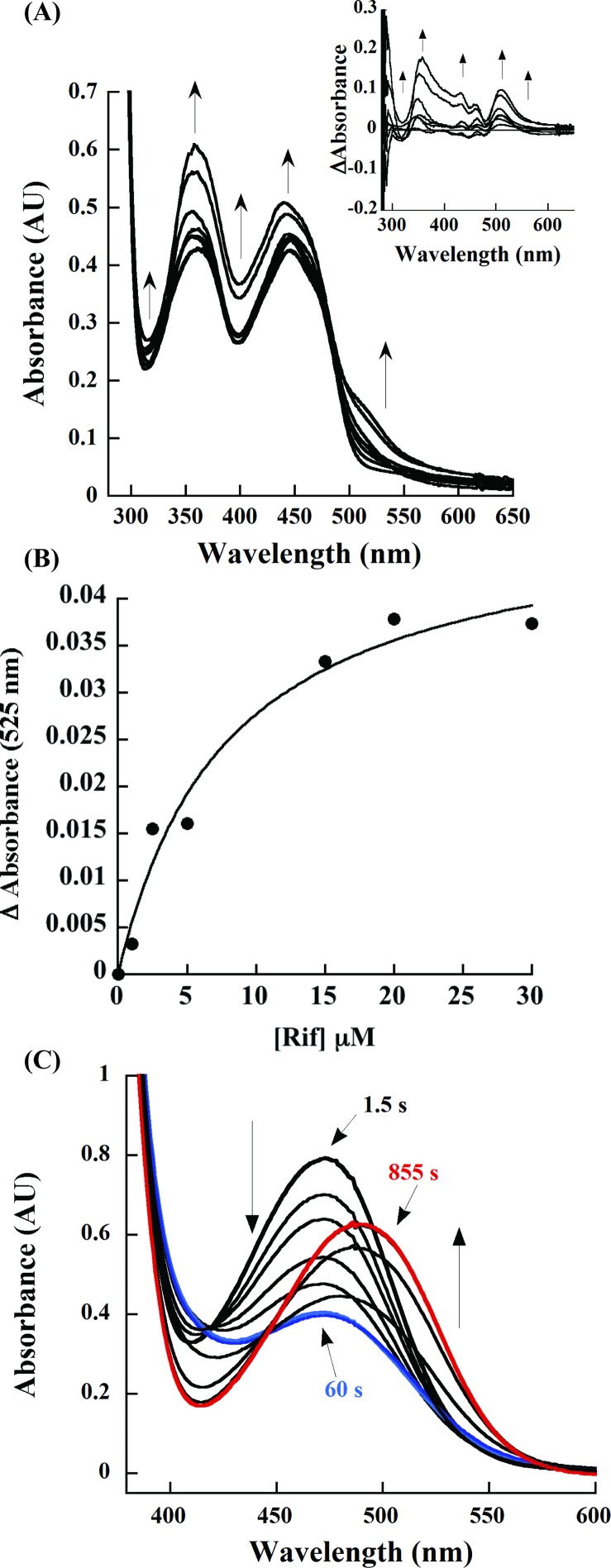
Spectral changes during Rif binding and turnover. (A) Flavin spectral changes as a function of increasing concentration of Rif (0–40 μM). The spectral changes show increases in absorbance at (320, 360, 400, 443, and 525 nm) and the isobestic point at ~ 480 nm. The inset shows spectral differences after subtracting the spectrum of RifMO with 0 μM Rif. (B) Determination of the *K*_D_ value of Rif. The change in absorbance of the RifMO-Rif complex at 525 nm was plotted as a function of Rif to determine a *K*_D_ value. (C) UV-Vis spectrophotometric monitoring of RifMO product formation representing the decline of the Rif peak at 475 nm (blue), followed by red shifting and an absorbance increase at 493 nm (red).

Rif binding was found to cause spectral changes to the bound FAD moiety of oxidized RifMO, as shown in (Fig 3A). The RifMO-Rif complex (ratio 1:1) caused an increase in absorbance along the FAD spectra with an isosbestic point at ~ 480 nm. The peak at 366 nm had a hypsochromic shift to ~ 354 nm (Fig 3A). The peak at 450 nm became broader and increased upon addition of Rif. Using spectral perturbation, the RifMO-Rif complex at different Rif concentrations was subtracted from the spectrum of unbound RifMO. The observed spectral changes showed increases at 320, 360, 400, 440, and 525 nm and were found to be substrate dependent. Changes in absorbance at 525 nm were used to determine a *K*_D_ value of 9.5 ± 2.5 μM for Rif (Fig 3B).

The UV-Vis spectrum of Rif in sodium phosphate buffer (pH 7.5) exhibited maximum absorbance at wavelengths of 237, 255, 334, and 475 nm (Fig 2B) [18]. Following the enzymatic oxidation of Rif in the visible range (400–600 nm), two species were observed. The first species was characterized by a decline in absorbance at 475 nm (up to 60 s), followed by a second species that was characterized by an absorbance increase and a shift to a longer wavelength (~493 nm). No further spectral changes were observed after 855 s (Fig 3C). This stepwise change implies the formation of two different reaction products, P* and Rif-OH.

### Oxygen consumption assay

**Fig 4:**
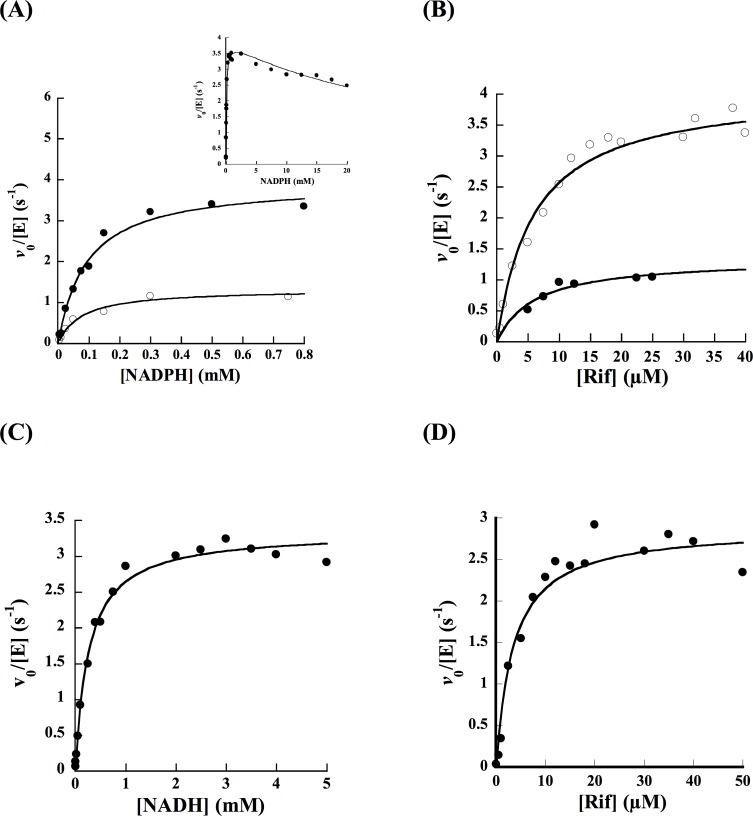
Steady-state kinetics of oxygen consumption compared to HPLC analysis. (A) Reaction rates as a function of NADPH using oxygraph (solid circles) and HPLC (open circles). The inset shows the oxygen consumption activity at higher NADPH concentration. (B) Reaction rates as a function of Rif using oxygraph (open circles) and HPLC (closed circles) in the presence of 1 mM NADPH as the electron donor. Oxygen consumption assays were done in 1 mL of 100 mM sodium phosphate, pH 7.5, at 25°C. (C) Oxygen consumption as a function of NADH. (D) Oxygen consumption as a function of Rif in the presence of 2 mM NADH as the electron donor.

Since molecular oxygen is incorporated into Rif to form Rif-OH, the change in oxygen concentration was monitored over time to determine the steady-state kinetic parameters with either NADPH or NADH (Table 1, Fig 4). RifMO was not very active in the absence of Rif (0.1 s^-1^), exhibiting ~20-fold lower activity with 0.5 mM NADPH compared to the reaction in the presence 50 μM Rif (2 s^-1^). Measuring the activity as a function of coenzyme concentration permitted the calculation of a *k*_cat_ value of 4.0 s^-1^, with either coenzyme, while the *K*_m_ value for NADPH was ~ 3-fold lower compared to NADH (Table 1). The catalytic efficiency for NAPDH was ~ 3.5-fold higher than for NADH. The *K*_m_ value for Rif was ~ 5 μM and the *k*_cat_/*K*_m_ value was 700,000 M^-1^s^-1^ with either NADH or NADPH (Table 1).

**Table 1:** Activity of RifMO monitored by oxygen consumption and HPLC.

Parameter	*k*_cat_ (s^-1^)	*K*_m_ (μM)	*k*_cat_/ *K*_m_ (M^-1^s^-1^)	*K*_I_ (mM)
**Oxygen consumption**				
**NADPH**	4.0 ± 0.1	90 ± 10	43,100 ± 3,700	33 ± 4.0
**Rif**	4.0 ± 0.16	5.8 ± 0.88	700,000 ± 81,300	--
**NADH**	3.30 ± 0.06	266 ± 25	12,600 ± 1,000	--
**Rif**	3.2 ± 0.1	4.5 ± 0.7	701,600 ± 88,100	--
**HPLC**				
**NADPH**	1.30 ± 0.05	75 ± 10	17,550 ± 2,650	--
**Rif**	1.3 ± 0.1	6.0 ± 1.5	222,400 ± 51,100	--
**Uncoupling (%)**	68			

Conditions: 100 mM sodium phosphate, pH 7.5, 25°C. When NADPH, NADH, and Rif were varied, saturating concentrations of 1 mM for NADPH, 2 mM for NADH, and 30 μM for Rif were used.

### Product formation assay

**Fig 5:**
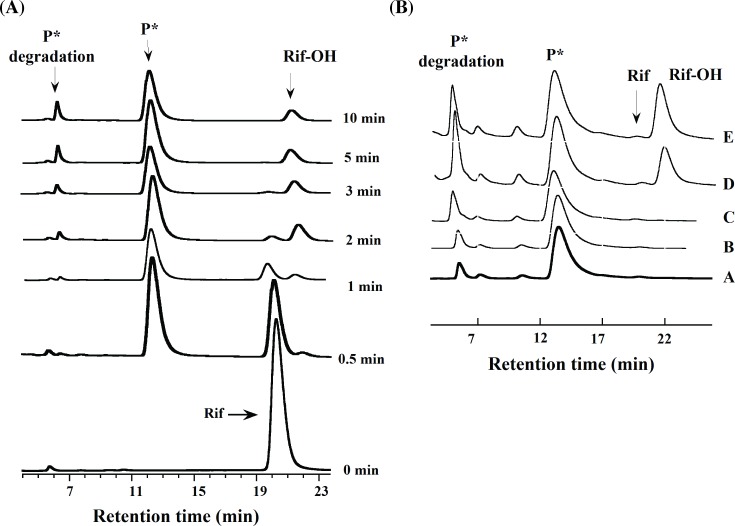
Time-dependent HPLC analysis of RifMO reactions. (A) Stacked chromatograms showing time traces for the elution of the Rif peak (21.2 min), P* (13.4 min), Rif-OH (22.1 min), and the P* degradation compound (6.7 min). (B) Stacked chromatograms show P* (A) extracted in 100 mM sodium phosphate buffer, pH 7.5, incubated with: (B) NADPH, (C) RifMO, (D, E) NADPH and RifMO, for 5, and 20 min., respectively.

The RifMO reaction was analyzed by HPLC in the presence of NADPH as the coenzyme. The analysis revealed two products, with the initial rate being calculated based on the formation of the first product (P*) (Fig 5). *K*_m_ values were similar to those determined with the oxygen consumption assay, but the catalytic efficiencies were lower due to lower *k*_cat_ values (Table 1, Fig 4A & 4B). By taking the ratio of the *k*_cat_ value calculated in the oxygen consumption assay and the *k*_cat_ value calculated in the HPLC assay, it was determined that the RifMO reaction is highly uncoupled (~ 68%).

### HPLC analysis of RifMO reaction product(s)

**Fig 6:**
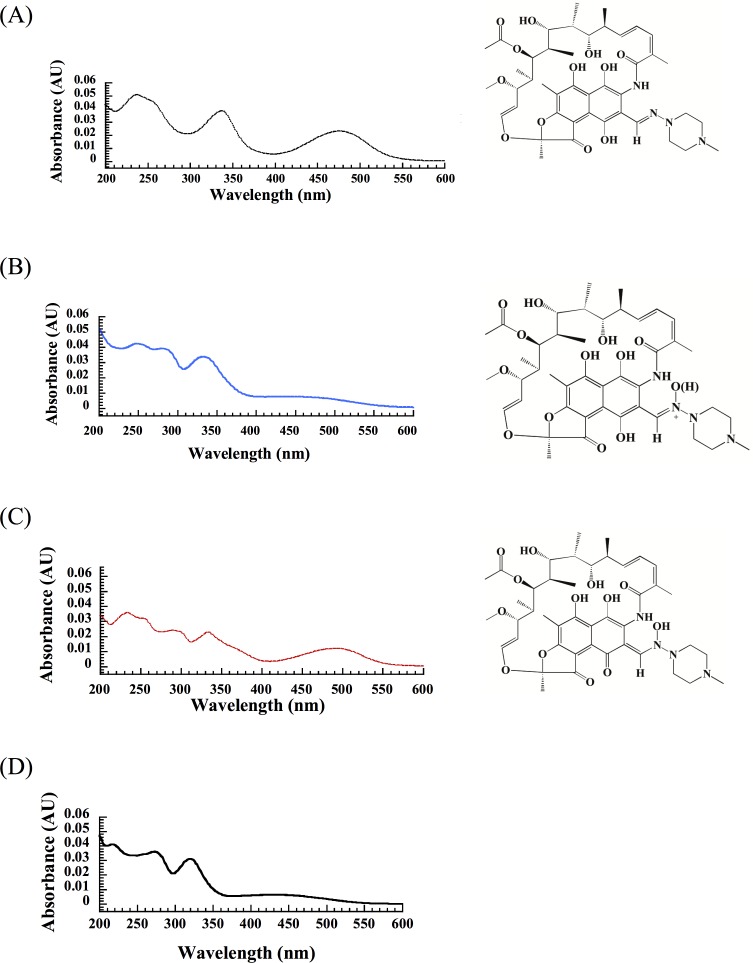
Individual UV-spectra extracted for pure peaks during HPLC analysis, representing all species involved in the RifMO reaction. (A) Rif, (B)The first product, (P*), (C) The final product, Rif-OH, and (D) Rif degradation compound of the first product. Rif-OH structure was elucidated from the NMR analysis.

Progress of the RifMO-catalyzed reaction was monitored by following the decrease of the Rif peak as well as the increase in the product peak(s) at 334 nm (Fig 5A). Rif (R_T_ = 21.2 min) was converted into two products: P* (R_T_ = 13.4 min) and Rif-OH (R_T_ = 22.1 min). Further analysis of RifMO activity, using purified P* (prepared from an enzymatic reaction) as the primary substrate, suggested that Rif-OH formation was enhanced in the presence of both RifMO and NADPH (Fig 5B). Additionally, another peak (R_T_ = 6.7 min) was observed at prolonged incubation reaction time (≥ 2 min) (Fig 5A), and this peak was found to be a degradation product of P*. Comparison of the UV-Vis spectra of the indicated species formed during the RifMO reaction suggested that they could have different chemical compositions (Fig 6). Furthermore, the observed spectral changes within the visible region (400–600 nm), suggests that the RifMO reaction is mostly associated with oxidation at the C1 and C4 atoms of Rif. As will be discussed later, these changes are associated with the hydroxylation of the N2′-atom that present within the piperazine group [19].

### Product(s) isolation and characterization

Flow-injection electrospray ionization mass spectrometry was used to characterize the products of the RifMO reaction. Samples were collected as individual peaks after HPLC separation as described in the methods section. The data showed a molecular ion peak [M+Na]^+^ at *m/z* 845.4 calculated for Rif in the presence of sodium phosphate in composition buffer. P* and Rif-OH showed [M+K]^+^ peaks at *m/z* 879.4 (HPLC elution buffer contained potassium ions). This result implies the addition of one oxygen atom to Rif to produce P*, which is subsequently converted into Rif-OH. The identical [M+K]^+^ peak value of both P* and Rif-OH is consistent with these compounds having the same molecular formula (C_43_H_58_N_4_O_13_), but are present in different isomeric forms.

**Fig 7:**
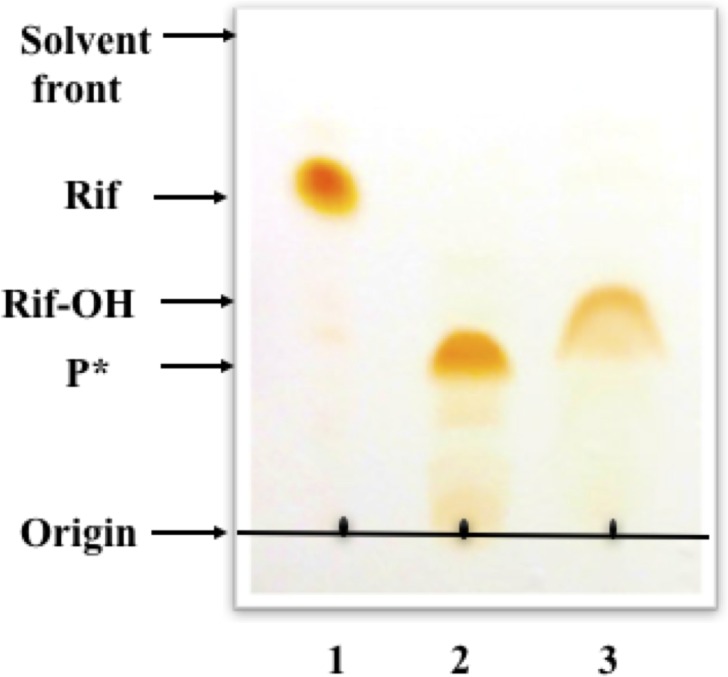
TLC monitoring of RifMO activity with Rif. Lane 1, Rif; lane 2, P*-generating reaction; lane 3, Rif-OH-generating reaction.

For further product characterization, large-scale reactions under two different conditions were used for the formation of only one of the products. Using different conditions was important to facilitate the extraction and purification as P* and Rif-OH separate with very similar R_f_ values using TLC (0.38 and 0.44, respectively) (Fig 7). Next, NMR was used for structural analysis of the TLC-purified Rif-OH. P1, however, was unstable over time and could not be used. Chemical shifts were assigned for ^1^H-NMR of Rif-OH (S1 Table). Compared to Rif, the chemical shift at the H_3_-34 signal (δ_H_ -0.27) was downfield shifted to (δ_H_ 0.7) in Rif-OH. A similar value (δ_H_ 0.69) has been reported for Rif-O (Rif-OH described here) by Hoshino *et al*. [11]. These data are also consistent with the downfield shift observed in the oxidation of rifamycin SV (naphthaquinol form) to rifamycin S (naphthaquinone form) [20].

### Flavin reduction

**Fig 8:**
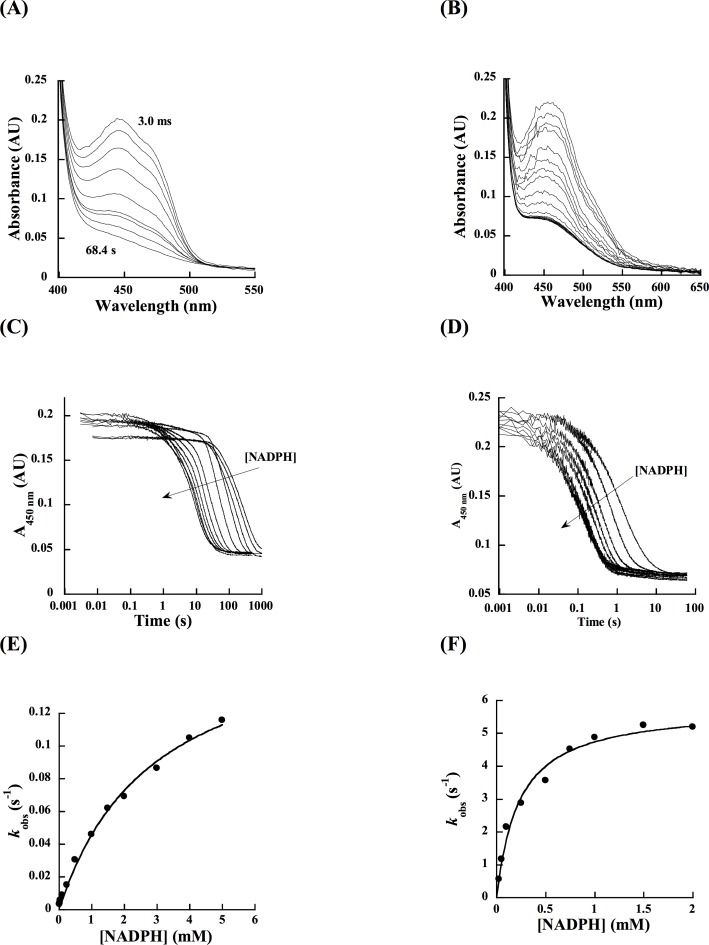
Flavin reduction with NADPH. Spectra changes for the substrate-free RifMO with 2 mM NADPH. (B) Change in the flavin absorbance at 450 nm for substrate-free RifMO at various concentrations of NADPH (0.025–2 mM). (C) Dependence of the *k*_obs_ values as a function of NADPH in the presence of 15 μM Rif. Data was fit to a single exponential decay equation. (D) Spectra changes for the Rif-RifMO complex with 2 mM NADPH. (E) Change in the flavin absorbance at 450 nm for substrate-complexed RifMO at various concentrations of NADPH (0.025–5 mM). (F) Dependence of the *k*_obs_ values as a function of NADPH in the absence of Rif. Data was fit to a single exponential decay equation.

The decrease in absorbance of the flavin cofactor at 450 nm as a function of NADPH concentration in the presence or absence of Rif were monitored in a stopped-flow spectrophotometer under anaerobic conditions (Fig 8). The data indicate a single NADPH-dependent reduction phase in both cases. Fitting the data to a single exponential decay equation revealed that the reduction process was significantly slower when Rif was absent; the rate constant for flavin reduction (*k*_red_) rate was enhanced ~ 30-fold in the presence of a stoichiometric amount of Rif. Furthermore, the *K*_D_ value for NADPH was lowered ~ 17-fold. Also, in the presence of Rif, the *k*_red_/ *K*_D_ value was ~550-fold higher than in the absence of Rif (Table 2). These data show that formation of the RifMO-Rif complex is crucial for FAD reduction.

**Table 2:** Rapid reaction kinetic parameters.

Parameter	No Rif	Rif
***k***_**red**_ **(s**^**-1**^**)**	0.20 ± 0.01	6.0 ±0.1
***K***_**D**_ **(mM)**	2.9 ± 0.3	0.17 ± 0.01
***k***_**red**_**/ *K***_**D**_ **(M**^**-1**^**s**^**-1**^**)**	62 ± 4.0	34,400 ± 1,900
***K***_**ox**_ **(M**^**-1**^**s**^**-1**^**)**	4,930 ± 840	5,600 ± 730

Conditions: 100 mM sodium phosphate, pH 7.5, 15°C. Reduction and oxidation of enzyme-substrate complex was done with 15 μM Rif.

The spectral changes observed during the reduction were different depending on whether Rif was present or not (Fig 8); both cases showed decreases in absorbance at 450 nm, which is an indication of FAD getting reduced, but the overall absorbance of FAD was enhanced by Rif binding as shown earlier in the UV-Vis spectrophotometric analysis for Rif binding.

### Flavin oxidation

**Fig 9:**
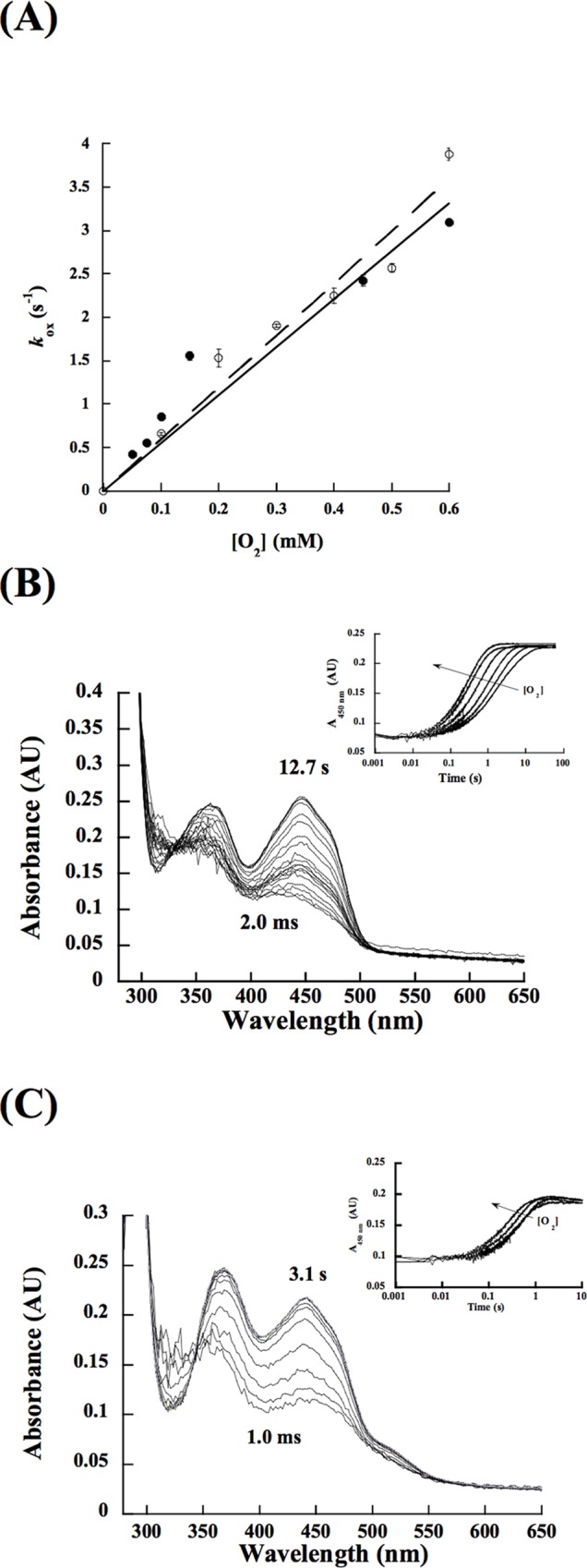
Flavin oxidation. (A) *k*_obs_ values as a function of [O_2_] without Rif (closed circles) and with 15 μM Rif (opened circles). (B) Spectra changes during the oxidation of free reduced RifMO with 250 μM O_2_. The inset shows the changes in absorbance at 450 nm as a function of oxygen concentration. (C) Spectra changes during the oxidation of free, reduced RifMO with 200 μM O_2_. The inset shows changes in absorbance at 450 nm as a function of oxygen concentration. Both data were fit to a single phase exponential equation.

The oxidation of reduced RifMO and the RifMO-Rif complex were monitored as a function of oxygen concentration in single and double mixing modes, respectively. In both experiments, the enzyme oxidation was monophasic, lacking the typical spectrum of stable C4a-(hydro)peroxyflavin species (Fig 9). The rate constants for oxidation were similar in the presence or absence of Rif (Table 2). This revealed that the half-oxidative reaction is not Rif-dependent, in contrast to the reduction step. It is worthwhile to mention that the peak at 525 nm starts to appear again with FAD re-oxidation within the RifMO-Rif complex, implying that substrate/product interaction with the enzyme is more pronounced in the oxidized state of RifMO.

## Discussion

Antibiotic resistance has been identified as one of the major health problems in modern medicine [21]. The mechanism of resistance can originate by mutation(s) of the target protein or enzyme, which leads to reduced antibiotic activity [10, 17, 21]. Soon after the discovery of Rif by Prof. Piero Sensi, reports describing the isolation and identification of Rif-resistant bacteria were published [3, 22–25]. The most common mechanism of Rif resistance occurs *via* mutations on the RNAP β-subunit (*rpoB2* gene); sequence analysis of resistant strains identified mutations in clusters that are known as the Rif resistant-determining region (RRDR) [16, 26, 27]. The structure of the RNAP β-subunit in complex with Rif showed that several of the reported RRDR contact Rif or are in close proximity [28]. In addition to mutations on the RNAP β-subunit, Rif resistance also occurs by enzymatic inactivation through chemical modification of Rif by glycosylation and phosphorylation reactions [7, 29, 30]. In addition, modification of Rif by hydroxylation has been reported to initiate Rif degradation in *N*. *farcinica*, this reaction was catalyzed by the product of the *rox* gene (which codes for RifMO) [11]. Previous studies revealed that introduction of the *rox* gene in a *Norcardia* strain lacking the *rpoB2* gene leads to elevated Rif resistance (32 fold) [11]. Thus, RifMO plays a role in Rif resistance in *N*. *farcinica*. RifMO related enzymes might also play a role in antibiotic resistance in *Mycobacteria sp*. as RifMO homologs are also present in these bacteria [9, 11]. Based on conservation of the FAD binding domain, RifMO was proposed to be a flavin-dependent monooxygenase, but phylogenetic analysis shows that it clusters separately from members of the Class A and B family of enzymes [11]. Recombinant RifMO was expressed as a soluble, stable, FAD-containing enzyme. Steady-state kinetic analysis showed that recombinant RifMO displays a 3-fold preference for NADPH over NADH (Table 1 and Fig 4). This is consistent with most members of the Class A monooxygenases such as *p*-hydroxybenzoate hydroxylase (PHBH) from *Pseudomonas fluorescens* and *Pseudomonas aeruginosa* PAO1, which are strictly NADPH-dependent, as well as the monooxygenase domain of human MICAL-1 (molecule interacting with CasL), which exhibits ~ 200-fold NADPH preference [31–35]. When activity was monitored by measuring the decrease in the concentration of molecular oxygen, the enzyme appeared to have very low activity when NAD(P)H was the only substrate present. However, when Rif was added the turnover rate increased ~ 20-fold. This increase in the activity upon substrate binding is also present in Class A and Class B enzymes [33, 36–38]. In Class A monooxygenases, binding of the substrate to be hydroxylated is required prior to the flavin reacting with NAD(P)H [39]. This phenomenon has been characterized in detail for PHBH, where upon binding of *p*-hydroxybenzoate (pHB), the hydroxyl group is deprotonated and the negative charge triggers the movement of the flavin from an “in position,” where the isoalloxazine ring is protected from the solvent, to an “out position,” where the flavin can react with NADP(H) [33]. The binding of pHB enhances the rate of flavin reduction by 10^5^-fold with no effect on the dissociation constant of NADPH [36]. This activation mechanism ensures that the enzyme, with reduced flavin, can react with molecular oxygen and forms the C4a-hydroperoxyflavin intermediate only when the substrate is present, thereby preventing production of H_2_O_2_ and NADP^+^ in an uncoupled reaction [36, 40]. In Class B monooxygenases, binding of the hydroxylatable substrate is not required for effective reaction with NAD(P)H. These enzymes prevent wasteful utilization of NAD(P)H and production of H_2_O_2_ by stabilizing the C4a-hydroperoxyflavin, utilizing a mechanism that requires NAD(P)^+^ to remain bound in the active site [37, 38, 41, 42]. This intermediate has been shown to have half-lives of up to ~ two hours in liver microsomal FAD-containing monooxygenases [43].

To investigate if the increase in RifMO’s oxygen consumption in the presence of Rif was due to turnover of the C4a-hydroperoxyflavin or activation of reactivity with NAD(P)H, flavin reduction in the stopped flow spectrophotometer was monitored under anaerobic conditions. In the absence of Rif, the rate constant for flavin reduction (*k*_red_) was very slow (Fig 8), which was consistent with the oxygen consumption rate measured without Rif in the presence of 0.5 mM NADPH. In the presence of an equimolar concentration of Rif, the *k*_red_ value increased ~30-fold. This increase in the *k*_red_ value matches the observed increase in the oxygen consumption assays and is consistent with the binding of Rif first priming the FAD for reaction with NAD(P)H, as described for Class A flavin-dependent monooxygenases. (Fig 8) [32, 36, 39]. This is also consistent with the binding of Rif to oxidized RifMO determined by monitoring the changes in flavin absorbance (Fig 3).

The reaction of reduced RifMO and the RifMO-Rif complex with molecular oxygen was also studied in the stopped-flow spectrophotometer. Binding Rif had no effect on flavin oxidation and no stable oxygenated flavin intermediates were observed during the time-resolved oxidation experiments (Fig 8). This is consistent with the 68% uncoupling determined by dividing the *k*_cat_ obtained from the oxygen consumption assay with that from the HPLC analysis (Table 1).

The chromatographic studies showed that two products were formed, P* and Rif-OH (Figs 5 and 6). P* appeared first, but due to its relative instability, it was difficult to use after purification for structural analysis by NMR. However, we were able to separate and collect both products P* (colorless) and Rif-MO (reddish) by HPLC for mass spectrometry analysis. Rif-MO formed second and its formation is accelerated in the presence of RifMO and excess NADPH (Fig 5B). Formation of the two products was initially monitored by the spectral perturbation of the Rif UV-Vis spectrum, which revealed a decrease in the absorbance of the peak at 475 nm (*i*.*e*., P* formation), followed by an absorbance increase with a red shift to 493 nm (*i*.*e*., Rif-OH formation) (Fig 3C). Fig 6 shows the UV-Vis spectra for each species, which is consistent with the observed spectral changes during the RifMO reaction.

To gain further insight into the nature of the extra product observed during HPLC analysis, we set two different conditions that allowed the production and purification of each product. The isolated products ran as two different spots on TLC plates (Fig 6)

Characterization of the product(s) of the RifMO reaction was consistent with Rif oxygenation. The final product (Rif-OH) is proposed to be 2′-N-hydroxy-4-oxo-Rif based on the NMR downfield chemical shift of the H_3_-34 atom signal (-0.27) in Rif to (0.7) Rif-OH (S1 Fig and S1 Table). This downfield chemical shift has been reported to be characteristic for the oxidation of a naphthaquinol moiety to naphthaquinone, which is the case here upon the hydroxylation of the N2′-atom of Rif to form Rif-OH (Fig 1) [11, 20]. Furthermore, the measured *m/z* value of 879.4 (calculated as [M+K]^+^) for the potassiated P* and Rif-OH was determined, suggesting that P* is a tautomer of Rif-OH. In addition, the rearrangement of the naphthaquinol ring (P*) to naphthaquinone (Rif-OH) are consistent with the spectral changes observed during the RifMO reaction (Figs 3 & 6).

**Fig 10:**
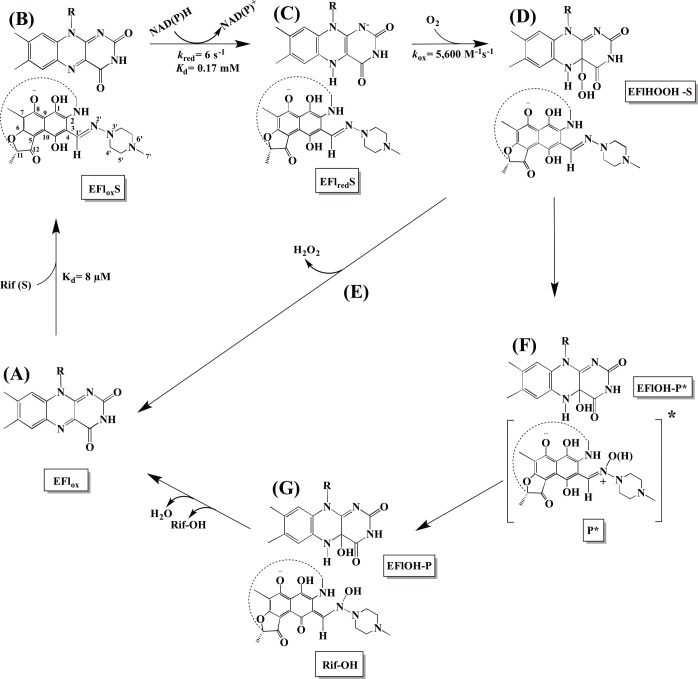
Catalytic cycle of RifMO. The reaction starts by the binding of Rif to oxidized RifMO (A), which primes the enzyme for the next step by inducing conformational changes in the flavin (B). NAPDH binds and reduces the flavin (C), and the reduced Rif-MO/Rif complex reacts with molecular oxygen to form the C4a-hydroperoxyflavin (D). This intermediate is not very stable and only ~30% hydroxylates Rif (F). The rest decays to hydrogen peroxide (E). The first product is the quinol (P*) (F), which is then converted to the final quinone product, Rif-OH (G). Release of Rif-OH and dehydration of the flavin are the final steps in the reaction.

Together the data is consistent with the catalytic cycle of RifMO depicted in Fig 10. The reaction is initiated by tight binding of Rif to the oxidized RifMO, which presumably induces movement of the flavin such that an effective reaction with NAD(P)H can take place as observed in other members of Class A flavin-dependent monooxygenases (Fig 10A and 10B) [31, 33, 39, 44, 45]. Unlike PHBH, in which the substrate deprotonation is initiated by the enzyme through a proton transfer network [33, 46], Rif deprotonation is not expected to be enzymatically-catalyzed since Rif is already present as a zwitterion with the 8-OH hydroxyl deprotonated at neutral pH [47, 48]. Mutation studies in the RifMO homolog PgaE (an aromatic hydroxylase involved in angucycline biosynthesis; 44% identity) confirmed that substrate deprotonation is enzyme-independent and is facilitated by substrate aromaticity [49]. RifMO complexed with Rif binds to NAD(P)H and reduces the flavin by the transfer of a hydride equivalent (Fig 10C). The reduced Rif-MO/Rif complex can react with molecular oxygen forming the C4a-hydroperyflavin intermediate, which is not stable and is only able to participate in Rif hydroxylation by ~ 30% (Fig 10C–10G). Later, the initial product (P*) bound to RifM is converted to Rif-OH by tautomerization (Fig 10G).

Flavin-monooxygenases are involved in catalysis of many reactions of environmental, industrial, and pharmaceutical importance such as the degradation of xenobiotics in bacteria and mammals and in the biosynthesis of natural products [50–52]. For example, siderophores, important virulence factors involved in iron acquisition, which is required for pathogenic metabolism and growth, are catalyzed by flavin monooxygenases [53–55]. Mainly, the flavin monooxygenases involved in the siderophore biosynthetic pathway are members of Class B, such as SidA from *Aspergillus fumigatus* and NbtG from *N*. *farcinica* [39, 56, 57]. In addition, it has been shown that other flavin-dependent monooxygenases, belonging to Class A, are involved in antibiotic resistance such as tetracycline monooxygenase (TetX) [58]. TetX from *Bacteroides fragilis* utilizes NADPH as a coenzyme to hydroxylate a variety of tetracycline antibiotic derivatives in their degradation pathways [58]. However, no detailed biochemical characterization is currently available for TetX. Similar tetracycline modifying enzymes are involved in tetracycline biosynthesis such as anhydrotetracycline hydroxylase (OxyS) [59]. Recently, a new class of tetracycline destructases, which also hydroxylate tetracycline, have been identified. These enzymes are predicted to be distinct from TetX enzymes, however, no biochemical characterization is currently available [60]. This paper presents the first detailed characterization of a flavin-monooxygenase involved in antibiotic degradation and resistance. These findings can provide insights for new strategies to overcome antibiotic resistance in *Nocardia sp*. and other related species.

## Materials and Methods

### Materials

NADPH and Rif were purchased from MP Biomedicals (Billerica, MA). NADH and NADP^+^ were purchased from Acros Organics (New Jersey, US) and FAD was purchased from Sigma (St. Louis, MO). All solvents were either reagent grade or high performance liquid chromatography (HPLC) grade. The concentrations of the following compounds were determined using their absorption coefficients at pH 7.50: NADPH ε_340_ = 6.27 × 10^3^ M^-1^ cm^-1^, Rif ε_475_ = 15.4 × 10^3^ M^-1^ cm^-1^, Rif ε_334_ = 28 × 10^3^ M^-1^ cm^-1^, product intermediate (P*) ε_334_ = 22.4 × 10^3^ M^-1^ cm^-1^_,_ NADH ε_340_ = 6.22 × 10^3^ M^-1^ cm^-1^, and FAD ε_450_ = 11.3 × 10^3^ M^-1^ cm^-1^.

### Protein expression and purification

The gene coding for *N*. *farcinica* IFM 10152 RifMO (*rox*) was codon optimized for expression in *Escherichia coli* and cloned into pET15b for expression as an N-terminal 6xHis tagged protein (GenScript USA Inc.). The plasmid was transformed into TurboCells^®^ chemically competent *E*. *coli* (Genlantis, San Diego, USA), and spread onto Luria-Bertani (LB) agar plates containing ampicillin (50 μg/mL). A single colony was inoculated into 50 mL LB broth containing ampicillin (50 μg/mL) and incubated overnight at 37°C. Aliquots of this culture (~ 8 mL) were used to inoculate six 1 L flasks of autoinduction terrific broth (TB) containing ampicillin (50 μg/mL) [61]. The cultures were incubated at 37°C for ~ 6 h with agitation (250 rpm), until the optical density at 600 nm (OD_600_) reached a value of ~ 4. The temperature was then decreased to 18°C and the cells were incubated overnight, harvested by centrifugation, rapidly frozen in liquid nitrogen, and stored at −80°C.

For protein purification, all of the following steps were performed at 4°C. A 60 g cell pellet was re-suspended in 180 mL of buffer A (25 mM HEPES, 300 mM NaCl, 10 mM imidazole, and 5% glycerol, pH 7.5). The cell suspension was incubated with constant stirring for 1 h in the presence of 100 μM FAD, 1 mM phenylmethanesulfonyl fluoride (PMSF), and 0.1 mL of DNase I, RNase, and lysozyme (25 μg/mL each). Cells were lysed by sonication at 70% amplitude with 5 s on, 10 s off intervals for 20 min, on ice. Cellular debris and insoluble materials were precipitated by centrifugation at 40,000 *xg* for 1 h. The resulting supernatant was loaded onto three in-tandem 5 mL HisTrap columns (GE Healthcare) previously equilibrated with buffer A. The sample was injected at a flow rate of 2 mL/min, and the columns were washed with buffer A. The enzyme was eluted with 150 mL of imidazole gradient in buffer A (10 to 500 mM Imidazole) at a flow rate of 2 mL/min. Fractions were analyzed by SDS−PAGE, and those containing 6x-His-RifMO were pooled and concentrated using an Amicon Ultra-15 centrifugal filter units (30,000 MWCO, from Millipore, MA, USA).

For cleavage of the His-tagged RifMO, 19.2 mg thrombin (95 NIH units/mg protein from bovine plasma; Sigma Aldrich) were added to the concentrated protein sample (~ 150 mg) and dialyzed in 2 L dialysis buffer (25 mM HEPES, 150 mM NaCl, 25 μM CaCl_2_, pH 7.5) overnight at 4°C, followed by additional incubation for 3 h at room temperature. The dialysate was loaded onto three in-tandem 5 mL HisTrap columns previously equilibrated with imidazole-free buffer A. The flow-through fractions containing His-tag free RifMO were collected, concentrated, and loaded onto a desalting column (HiPrep, GE Healthcare) for exchange to buffer C (25 mM HEPES, pH 7.5). The desalted protein fractions were then loaded onto a DEAE column pre-equilibrated with buffer C, and 200 mL of a gradient of (0 to 500 mM NaCl in 25 mM HEPES, pH 7.5) was used for elution. Eluted fractions containing RifMO were pooled and dialyzed in 1L of storage buffer (100 mM sodium phosphate buffer, 50 mM NaCl, pH 7.5) for 8 h, followed by 2 h dialysis in 1 L fresh storage buffer. RifMO was concentrated to 10 mg/mL with the addition of glycerol to a final concentration of 20%. Aliquots were flash-frozen in liquid nitrogen prior to being stored at −80°C. Final protein sample purity was estimated to be ~ 95% based on SDS-PAGE.

### Determination of flavin incorporation and extinction coefficient

The flavin spectrum of purified RifMO (~ 30 μM) in 100 mM sodium phosphate buffer (pH 7.5), containing 50 mM NaCl and 20% glycerol was recorded in the protein bound and free states. Prior to monitoring the UV-Vis spectrum of FAD in the protein-free state, the flavin was extracted after denaturation at 95°C for 10 min, followed by centrifugation. The extinction coefficient of the FAD bound to RifMO was calculated to be 10,990 M^−1^ cm^−1^ at 450 nm based on the extinction coefficient at 450 nm for free FAD (11,300 M^−1^ cm^−1^). Flavin incorporation was determined by dividing the concentration of RifMO, calculated using flavin absorbance at 450 nm, by the concentration of enzyme, calculated using the Bradford assay with bovine serum albumin (BSA) as the standard.

### Rif binding to oxidized RifMO and spectral changes during catalysis

Binding of Rif to oxidized RifMO was monitored by recording the change in the flavin spectrum on an Agilent 8453 diode array spectrophotometer (Agilent Technologies, Santa Clara, CA, USA) as a function of increasing concentration of Rif. Each solution consisted of 200 μL of 100 mM sodium phosphate buffer (pH 7.5), RifMO (15 μM), and various Rif concentrations (0–40 μM). The solutions were incubated on ice for 10 min before data acquisition. The spectrum of each Rif concentration alone was subtracted from the spectrum of its corresponding Rif-RifMO complex. The spectral changes showed the appearance of a new peak at 525 nm. Additionally, absorbance values of each Rif-RifMO complex spectrum were subtracted from the spectrum of the free enzyme; absorbance differences at 525 nm were plotted as a function of Rif concentration to calculate the *K*_D_ value.

The reaction of RifMO was monitored by recording absorbance changes of the Rif spectrum in the visible range between 400 and 600 nm. In a 0.5 mL reaction volume, 50 μM Rif was mixed with 1 mM NADPH in 100 mM sodium phosphate buffer (pH 7.5) at 25°C. Spectra changes were recorded after addition of 1 μM RifMO. All spectra were corrected for background absorbance at 800 nm. Two distinct stages of the reaction could be observed. First a decrease in absorbance at 475 nm was observed, followed by a second state that consisted of a red shifted increase in absorbance to 493 nm. The stages have been proposed to be correlated with the formation of an intermediate product (P*) and the final product, Rif-OH.

### Oxygen consumption assay

A Hansatech Oxygraph (Norfolk, U.K.) was used to monitor the oxygen consumption rates by RifMO in the presence of Rif with NADPH or NADH. A total reaction volume of 1 mL of 100 mM sodium phosphate buffer (pH 7.5) was used. NADPH, NADH, and Rif concentrations were varied in the range of 5–1000 μM, 5–5000 μM, and 0–50 μM, respectively. For reactions where the variable substrate was NADPH or NADH, Rif was kept constant at 30 μM. For reactions where Rif varied, NADPH or NADH were kept constant at a concentration of 1 mM and 2 mM, respectively. The reaction proceeded for 1 min at 25°C with constant stirring. The assays were initiated by addition of 0.1 μM Rif-MO.

### HPLC analysis of RifMO reaction product(s)

Product(s) of the RifMO reaction were separated using a Shimadzu HPLC (prominence UFLC) system equipped with a photodiode array detector (PDA). Separation was achieved with a C18 reversed-phase column (Phenomenex Luna^®^ 100 Å, 250 x 4.6 mm, 5 μm) using an isocratic mobile phase that consisted of 65% of 10 mM potassium phosphate buffer (pH 7.5) and 35% acetonitrile. Samples (50 μL) were injected at a flow rate of 1 mL/min and each run proceeded for 25 min at 25°C. All HPLC-analyzed reactions were quenched with acetonitrile (35% final concentration); organic inactivation is better at quenching the reactions because it is non-destructive to Rif and its product(s). Protein was removed by centrifugation at 13,000 rpm for 2 min. Substrate and product(s) were monitored and quantified at 334 nm.

### Product formation assay

The formation of Rif-OH by RifMO was monitored and quantified by HPLC as described above. The reactions were performed in 0.2 mL of 100 mM sodium phosphate buffer (pH 7.5). Reactions were incubated in the presence of various concentrations of Rif (5–30 μM) or NADPH (5–1000 μM). When not varied, Rif and NADPH were kept constant at 30 μM and 1 mM, respectively. The reactions were initiated by the addition of 0.1 μM RifMO and incubated at 25°C for 30, 40, or 60 s. Each reaction was quenched, processed, and eluted as described in greater detail below. P* was quantified by measuring the peak area at 334 nm.

### Product(s) isolation and characterization

Two different reaction conditions were used to optimize the production and isolation of P* and Rif-OH to facilitate their characterization. Both reactions were incubated in 100 mM sodium phosphate buffer, pH 7.5, in the presence of an NADPH-regenerating system. For the P*-generating reaction, a 220 mL solution that consisted of 100 μM NADP^+^, 10 mM glucose-6-phosphate (G6P), and 500 units of glucose-6-phosphate dehydrogenase (G6PDH) was incubated at 25°C for 45 min with occasional shaking to generate NADPH. Next, 100 μM Rif was added to this mixture and the reaction was initiated by the addition of 1 μM RifMO and incubated for 10 min with shaking at 25°C. For the Rif-OH-generating reaction, a 20 mL solution containing 1 mM NADP^+^, 10 mM G6P, and 500 units of G6PDH was incubated at 25°C for 45 min with occasional shaking. Then, 1 mM Rif was added to this mixture and the reaction was initiated by the addition of 10 μM RifMO and incubated for 2.5 h at 25°C with shaking. The progress of each reaction was monitored by HPLC to validate the formation of only P* (Peak at 13.4 min, Fig 5) or Rif-OH (Peak at 22.1 min, Fig 5). The reactions were then quenched with equal volumes of chloroform. Upon phase separation, the lower layers were separated and the solvent was completely evaporated. Finally, the residual products were re-dissolved in methanol and used for subsequent purification and separation by preparative thin-layer chromatography (TLC), using 0.5 mm thick silica gel 60F_254_ (MP Biomedicals) and using the lower phase of the mixture [chloroform: methanol: H2O (65:15:5, v/v/v)] as the mobile phase as described previously [11]. The bands corresponding to P* and Rif-OH were scraped from the TLC plates and extracted with chloroform under sonication and centrifuged. The organic solvent was evaporated using a Rotavapor R-210 (BUCHI, Switzerland) at 100 mbar and 25°C until complete dryness. The dried samples were stored in a dark environment at 4°C for further analysis with nuclear magnetic resonance (NMR).

### Monitoring product(s) formation

To monitor the progress of the RifMO reaction as a function of time, a total reaction volume of 0.2 mL in 100 mM sodium phosphate buffer, pH 7.5, containing final concentrations of 500 μM NADPH, 100 μM Rif, and 1 μM RifMO was used. The reaction was initiated by the addition of the enzyme and allowed to incubate for (0.5, 1, 2, 3, 5, and 10 min) at 25°C. At the interval times, reactions were quenched and processed as described above. After injection, all the reaction components, including NADPH/NADP^+^, Rif, and reaction product(s) were monitored at 334 nm.

The conversion of P* to Rif-OH by RifMO was investigated over prolonged reaction incubation times. P* was produced and separated as described above with minor modifications. The P* corresponding band was directly scrubbed from the TLC plate, re-suspended in 100 mM sodium phosphate buffer (pH 7.5), and used immediately as a substrate for the RifMO reaction. Purified P* was used to run a series of reactions where it was incubated with 500 μM NADPH or 1 μM RifMO for 10 min as the controls, and with both 500 μM NADPH and 1 μM RifMO for 5, 10, and 20 min at 25°C as the test. Samples were quenched, processed, and eluted as described above.

### Mass spectrometry (MS)

Rif (10 μM, in sodium phosphate buffer, pH 7.5) and reaction product samples (collected during HPLC elution) were analyzed with an AB Sciex 3200 Q TRAP mass spectrometry system. The liquid chromatography system was an Agilent 1200 Series. Samples were injected into the mass spectrometer ion source using the flow injection technique. This method employs an Agilent auto-sampler to inject an aliquot from the selected vial into the mass spectrometer without using an HPLC column. The solvent system employed was 50% Barnstead Nanopure water with 0.1% ammonium acetate and 50% methanol. The flow rate was 0.5 mL/min. Electrospray ionization was employed at 4500 volts and a temperature of 600°C. Curtain gas, gas 1, and gas 2 flow pressures were 35, 70, and 60 psi, respectively. Desolvation, entrance, and collision cell entrance potentials were 40, 12, and 22.5 volts, respectively. The mass analyzer system was employed in the Enhanced Mass Spectrum mode, which uses the linear ion trap function, in this case to scan the mass range from 150–1000 Da at a rate of 1000 Da/sec. Mass spectral data was acquired for one minute before another sample was injected.

### NMR analysis

NMR was performed only for the final purified Rif-OH sample (extracted using preparative TLC as described above) because P* degrades over time. 2 mg of Rif-OH was dissolved in DMSO-d_6_ and the ^1^H-spectra were recorded on a Brucker Avance 600 instrument located in the Department of Chemistry, Virginia Tech. The chemical shifts are given in δ (ppm), and coupling constants (J) are reported in Hz.

### Flavin reduction

The reaction of NADPH with oxidized RifMO was monitored in the absence or presence of Rif at 15°C in 100 mM sodium phosphate buffer (pH 7.5), using a stopped-flow spectrophotometer (Applied Photophysics, UK) in single mixing mode. The stopped-flow spectrophotometer system and buffers were made anaerobic as described previously [57]. Oxidized RifMO (15 μM, after mixing) was mixed with various concentrations of NADPH in the range of (0.025–5 mM, after mixing) without Rif or (0.025 mM–2 mM, after mixing) in the presence of 15 μM Rif, respectively. Reactions were monitored with a photodiode array spectrophotometer until flavin reduction was completed.

### Flavin oxidation

The half-oxidative reaction was monitored in the absence or presence of Rif at 15°C in 100 mM sodium phosphate buffer (pH 7.5), in single and double mixing mode, respectively. Oxygen saturated buffer (1.2 mM) was obtained by bubbling 100% oxygen gas into a closed vial for 30 min at 25°C [62]. Various concentrations of oxygen (100–600 μM) were obtained by mixing 100% oxygen saturated buffer with anaerobic buffer.

When Rif was absent, oxidized RifMO (60 μM) was first reduced with NADPH (90 μM; 1.5-fold). This mixture was allowed to reduce inside the glove box for 75 min under anaerobic conditions. Reduced RifMO (15 μM, after mixing) was then mixed with the oxygenated buffer. For the oxidation of Rif-complexed enzyme, double mixing stopped-flow experiments were performed. In the first mixing step, 60 μM Rif-complexed enzyme (30 μM, after mixing) was allowed to react with a stoichiometric concentration of NADPH (30 μM, after mixing) for 30 s. In the second mixing step, reduced Rif-complexed enzyme was reacted with various concentrations of molecular oxygen (100 − 600 μM, after mixing). In both cases, the reactions were monitored with a photodiode array spectrophotometer until complete FAD oxidation was observed.

### Data analysis

The kinetic data were fit using KaleidaGraph (Synergy Software, Reading, PA). Steady-state kinetic data were fit to the Michaelis-Menten equation. Flavin reduction data at 450 nm was fit to a single exponential decay equation (Eq 1) and the resulting *k*_obs_ values were plotted as a function of NADPH concentration to determine the maximum rate of flavin reduction (*k*_red_) and the *K*_D_ values (Eq 2). Flavin oxidation data at 450 nm was fit to a single exponential rise equation (Eq 3). The resulting *k*_obs_ values were plotted as a function of oxygen concentration and fit to a linear equation for determination of the bimolecular rate constant for flavin oxidation (*k*_ox_).

$$v=C+A_{1}e^{-\left(k_{obs1}t\right)}$$ (1)

$$k_{obs}=\frac{k_{red}\times [S]}{K_{D}+[S]}$$ (2)

$$v=C+A_{1}({1-e}^{-(k_{obs}t)})$$ (3)

## Supporting Information

S1 Fig Comparison of ^1^H NMR spectrum of Rif (substrate) and Rif-OH (product). (PDF)

S1 Table ^1^H-NMR data for Rif-OH in DMSO-d_6_ (δ [ppm], *J* [Hz]). (PDF)
