# Supplementary figures and images for: Mechanism of Rifampicin Inactivation in Nocardia farcinica
Source: PLoS One. 2016 Oct 5;11(10):e0162578. doi: 10.1371/journal.pone.0162578 (PMC5051949; doi:10.1371/journal.pone.0162578)

**S1 Figure. Comparison of  $^1\text{H}$  NMR spectrum of Rif (substrate) and Rif-OH (product).**

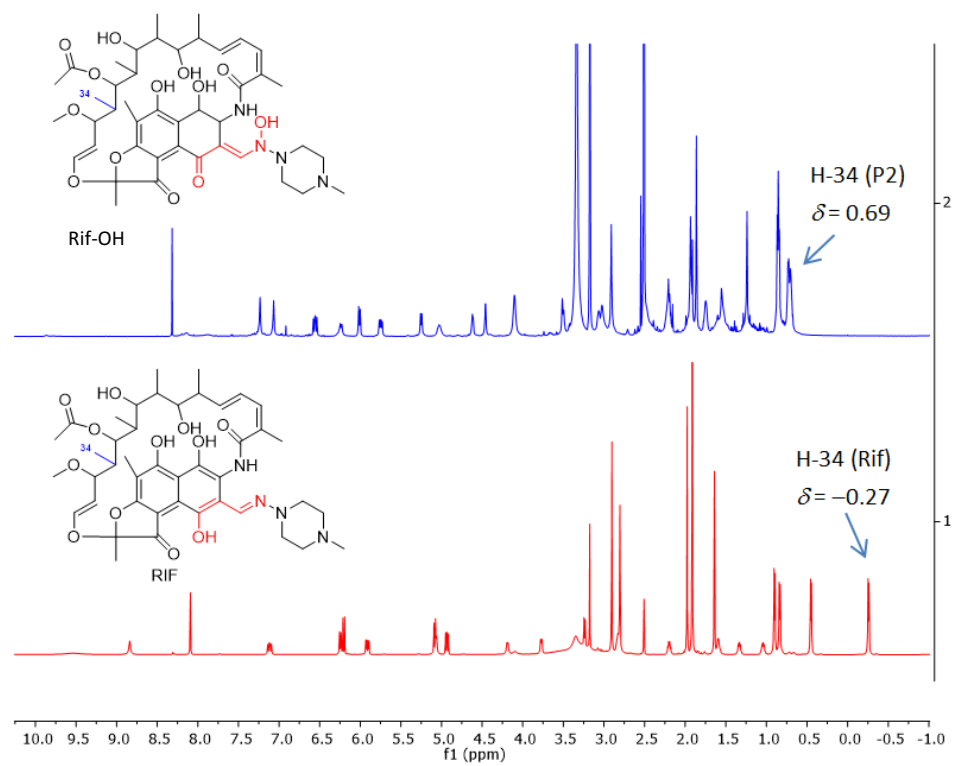

Supplement: S1 Fig — (PDF) [file pone.0162578.s001.pdf]
