# Supplementary material for: Mechanism of Rifampicin Inactivation in Nocardia farcinica
Source: PLoS One. 2016 Oct 5;11(10):e0162578. doi: 10.1371/journal.pone.0162578 (PMC5051949; doi:10.1371/journal.pone.0162578)
Supplement: S1 Table — (PDF) [file pone.0162578.s002.pdf]

S1 Table. ^{1}H-NMR data for Rif-OH in DMSO-d_6 (δ [ppm], *J* [Hz]).

	Data published[*]	Data obtained
position	δH (J in Hz)	δH (J in Hz)
13	1.63, s	1.63, s
14	2.09, s	2.15, s
17	6.00 , d (11.0)	6.01, d (10 .9)
18	6.55, dd (15.4, 11.0)	6..56, dd (15.3, 10.9)
19	5.74, dd (15.4, 7.7)	55.75, dd (15.4, 7.8)
20	2.18, m	2.20. m
21	3.48, m	3.51, m
OH-21	4,44, brd (3.3)	4,46, brd (3.7)
22	1.72, m	1.75, m
23	3.05, m	3.02, m
OH-23	4,59, d (6.6)	4,61, d (5. 6)
24	1.72, m	1.75, m
25	5.23 , d (10.1)	5.25, d (10 .1)
26	1.57, m	1.54, m
27	3.34, dd (8.5, 1.9)	3.35, m
28	5.1, dd (12.1, 8.5)	5.03, m
29	6.3, d (12.1)	6.24, d (12 .4)
30	1.85, s	1.86, s
31	0.83, d (6.6)	0.84, d (7.4)
32	0.84, d (6.8)	0.86, d (7.0)
33	0.71, d (6.8)	0.73, d (6.0)
34	0.69, d (6.8)	0.70, d (6. 8)
36	1.92, s	1.94, s
37	2.90, s	2.91, s
1'	8.28, s	8.32, s
4'	3.08, t (4.7)	3.07, m
5'	2.52, m	2.50, m (overlapped)
7'	2.24, s	2.22, s
NH	7.06 (0.5H), s	7.07, s
	7.23 (0.5H), s	7.23, s

[*] *Data obtained from reference [11]*
